# Role of angiotensin II in aging

**DOI:** 10.3389/fnagi.2022.1002138

**Published:** 2022-12-02

**Authors:** Wenmin Yi, Fei Chen, Huiji Zhang, Peng Tang, Minghao Yuan, Jie Wen, Shengyuan Wang, Zhiyou Cai

**Affiliations:** ^1^Department of Neurology, Chongqing Medical University, Chongqing, China; ^2^Chongqing Institute Green and Intelligent Technology, Chinese Academy of Sciences, Chongqing, China; ^3^Chongqing School, University of Chinese Academy of Sciences, Chongqing, China; ^4^Department of Neurology, Chongqing General Hospital, Chongqing, China; ^5^Chongqing Key Laboratory of Neurodegenerative Diseases, Chongqing, China; ^6^Department and Institute of Neurology, Guangdong Medical University, Zhanjiang, Guangdong, China

**Keywords:** aging, angiotensin II, reactive oxygen species, mitochondrial dysfunction, telomere attrition

## Abstract

Aging is an inevitable progressive decline in physiological organ function that increases the chance of disease and death. The renin–angiotensin system (RAS) is involved in the regulation of vasoconstriction, fluid homeostasis, cell growth, fibrosis, inflammation, and oxidative stress. In recent years, unprecedented advancement has been made in the RAS study, particularly with the observation that angiotensin II (Ang II), the central product of the RAS, plays a significant role in aging and chronic disease burden with aging. Binding to its receptors (Ang II type 1 receptor – AT_1_R in particular), Ang II acts as a mediator in the aging process by increasing free radical production and, consequently, mitochondrial dysfunction and telomere attrition. In this review, we examine the physiological function of the RAS and reactive oxygen species (ROS) sources in detail, highlighting how Ang II amplifies or drives mitochondrial dysfunction and telomere attrition underlying each hallmark of aging and contributes to the development of aging and age-linked diseases. Accordingly, the Ang II/AT_1_R pathway opens a new preventive and therapeutic direction for delaying aging and reducing the incidence of age-related diseases in the future.

## Introduction

During the past century, advances in modern medicine knowledge and improvements in examination technology have significantly augmented life expectancy (Kontis et al., 2017; Kalache et al., 2019). The global population, aged 60 years and above, is estimated to increase from 841 million in 2013 to approximately 2 billion in 2050 (Foreman et al., 2018). This demographic mile-stone will usually be accompanied by the high incidence of some diseases, such as cardiovascular disease, metabolic disorders, cancer, and neurodegenerative disorders (Lopez-Otin et al., 2013; Guzman-Castillo et al., 2017; Melzer et al., 2020), the incidence of which doubles every 5 years for people aged over 60 years. Aging is an irreversible and intricate process characterized by a generalized and time-dependent biological functional impairment, accompanied by genomic instability, telomere attrition, mitochondrial dysfunction, and cellular senescence (Lopez-Otin et al., 2013). Aging results in decreasing quality of life and has gradually become one of the major socioeconomic challenges of our era.

For the last few decades, the involvement of the renin–angiotensin system (RAS) in mediating vasoconstriction, ion entry and excretion, fibrosis, inflammatory and oxidative stress has been well documented (Benigni et al., 2009, 2010; Forrester et al., 2018). In addition to the circulating system, elements of the RAS are also found in diverse tissues of the brain, heart, and kidney, and contribute to the aging of these organs (Feng et al., 2011; Zablocki and Sadoshima, 2013; Forrester et al., 2018; Mogi, 2020; Labandeira-Garcia et al., 2021). Within the brain, different components of the RAS have been extensively studied in the context of neuroprotection and cognition (Jackson et al., 2018). Alterations in the brain RAS during aging may establish a link between impairment of autonomic reflex function and metabolic changes in aging (Diz et al., 2007). Further evidence for the relevance of the RAS in aging is derived from numerous experiments *in vivo* and *in vitro*, indicating that aging is accompanied by the increased activity of angiotensin II (Ang II), which is the major bioactive peptide of this system (Benigni et al., 2009; Salazar, 2018; Okuno et al., 2020). A prospective observational study (Benigni et al., 2009) effectively proved that the lifespan of mice with disrupted Ang II type 1 receptor (AT_1_R) was remarkably longer than that in the control group and showed that the mice lacking AT_1_R exhibited less oxidative damage, consequently indicating that Ang II-induced reactive oxygen species (ROS) by AT_1_R seemed to play a crucial part in the aging process. Considering that mitochondria are a key source of endogenous ROS (Cadenas, 2004; Balaban et al., 2005) and telomeres are particularly vulnerable to oxidative stress (Ahmed and Lingner, 2018; Barnes et al., 2019), in this article, we first describe the diverse components of the RAS as well as their physiological functions coupling with each other. Then, we provide an overview of the primary ROS sources and the mechanistic association of ROS with mitochondria and telomeres. This is followed by the discussion of the seemingly universal roles of mitochondrial dysfunction and telomere attrition in aging and how Ang II influences them, conducing to present new preventive strategies in fighting aging and age-associated diseases.

## General overview of the renin–angiotensin system

### Renin–angiotensin system peptides

In 1898, Robert Tigerstedt and Per Gunnar Bergman discovered a powerful vasopressor in the renal cortex, named rennin (Tigerstedt and Bergman, 1898). Since then, the RAS has been widely researched. The RAS was initially considered a circulating hormonal system that played a major role in the regulation of body fluid homeostasis through the control of blood volume and peripheral resistance. It was generally thought that the RAS appeared only in the classic circulatory system (Kinoshita et al., 2009). Later on, however, the local or paracrine RAS was identified in various tissues, including the brain, heart, and kidney (Stoll et al., 1995; Vaughan et al., 1995; Lee et al., 2010; Salgado et al., 2010; Pan et al., 2014; Labandeira-Garcia et al., 2021). The elements of the RAS can be cleaved during circulation by a series of enzymes and delivered to and expressed in its numerous target tissues or cells. The local tissue RAS contains two forms: intracellular and extracellular (Zablocki and Sadoshima, 2011; Zhuo and Li, 2011; Chappell et al., 2014), which further increases the difficulty of studying the RAS. The synthesis of intracellular RAS components and different types of RAS receptors is observed in some cells, including vascular smooth muscle cells, fibroblasts, cardiomyocytes, renal cells, and neurons (Re and Cook, 2015; Li et al., 2018; Re, 2018; Escobales et al., 2019). Recently, researchers have paid more attention to the role of the local RAS in specific tissues, such as the brain, the heart, and the kidney (Dzau and Re, 1994). The local paracrine RAS in the brain has been associated with several brain disorders, including Parkinson's disease (PD) (Costa-Besada et al., 2018). Several studies have suggested that the circulating RAS and tissue paracrine RAS may act together in peripheral tissues and that the two forms of the RAS are deemed to work in a complementary way, especially in the heart and the kidney (Lee et al., 2010). For example, the tissue RAS in the kidney regulates, together with the circulating RAS, not only renal cell growth and production of glomerulosclerosis but also blood pressure (Kagami et al., 1994; Ruiz-Ortega and Egido, 1997).

**Figure 1:**
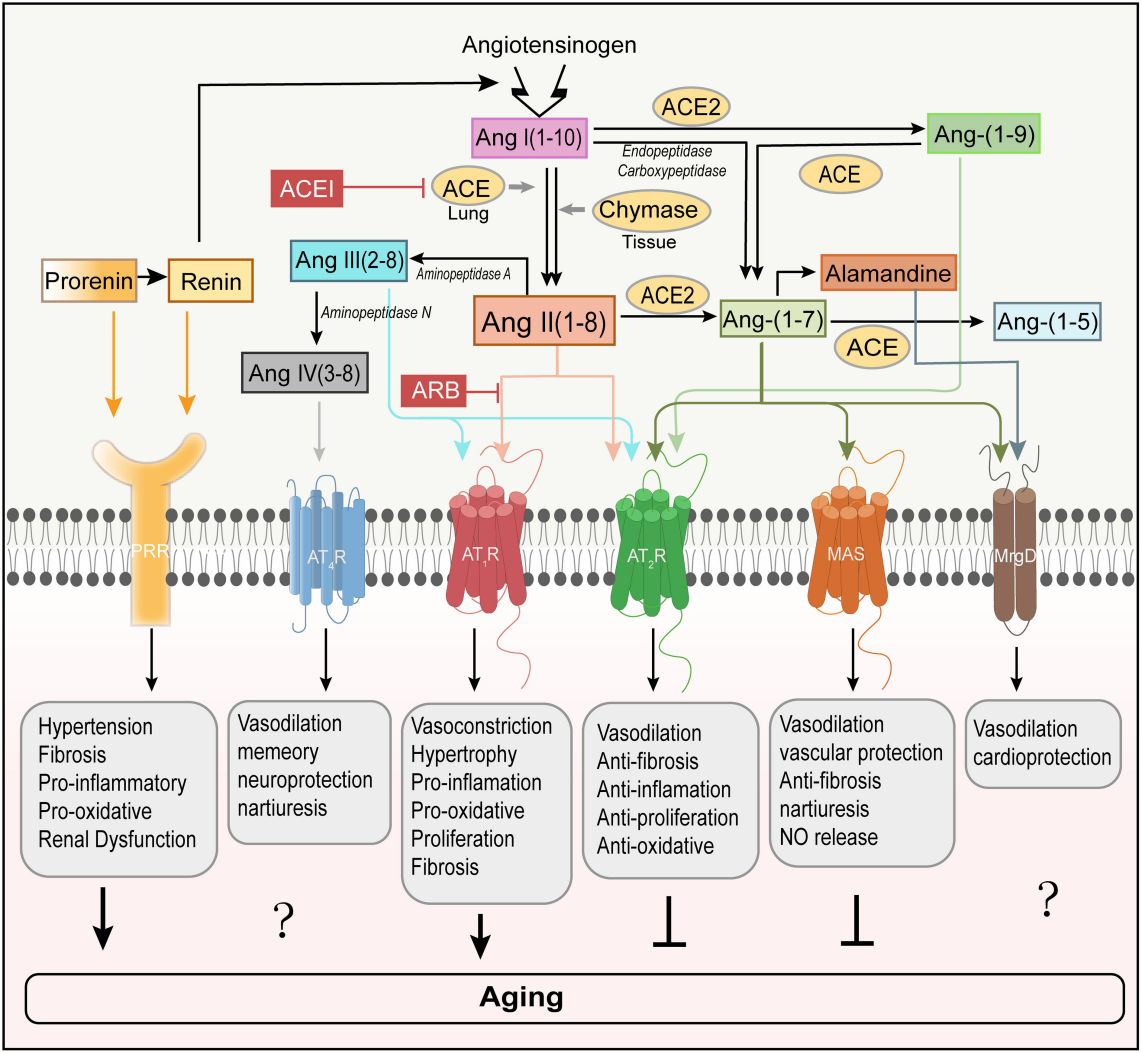
Involvement of RAS peptides and receptors in aging regulation. The classical RAS positively regulates the induction of aging by activating AT_1_R. In contrast, AT_2_R is regarded as a counterbalance to AT_1_R-mediated aging. PRR is a new member of the RAS receptors and mediates aging (Yoshida et al., 2019). Ang-(1–7) binds to MAS to counter-regulate of AT_1_R-mediated aging. However, the roles that Ang-(1–7) receptor MrgD and Ang IV receptor AT_4_R play in aging remain unclear. ACE, angiotensin-converting enzyme; ACE2, angiotensin converting enzyme 2; ACEI, angiotensin-converting enzyme inhibitor; ARB, angiotensin receptor blocker; AT_1_R, angiotensin type I receptor; AT_2_R, angiotensin type 2 receptor; AT_4_R, angiotensin type 4 receptor; MrgD, Mas-related-G protein-coupled receptor; PRR, (pro)renin receptor; NO, nitric oxide.

The renin–angiotensin system comprises numerous peptides and enzymes, including renin, an acidic protease secreted by juxtaglomerular cells, angiotensinogen, and different types of angiotensin and their receptors (Figure 1). Angiotensinogen is cleaved by renin to form angiotensin I (Ang I), a decapeptide with weak biological activity. Ang I is subsequently hydrolyzed by angiotensin-converting enzyme (ACE) to form octapeptide Ang II, a hormone that has strong hemodynamic effects (Boehm and Nabel, 2002). Ang II can undergo further hydrolysis into angiotensin III (Ang III) and angiotensin IV (Ang IV) by other aminopeptidases in plasma and tissue. ACE is primarily membrane bound on the endothelium, mainly in the epididymis, testes, and lungs, and it is shed in the plasma. The ACE gene lies on human chromosome 17q23. Genetic analysis has uncovered that ACE I/D polymorphism, involving a 287 bp DNA repeat sequence that is inserted(I) or deleted (D) in intron 16 of chromosome 17, accounts for about 50% of the total phenotypic variance of ACE (Zhu et al., 2012). Studies on all the primary races have suggested that the ACE I/D polymorphism is associated with systemic lupus erythematosus, type 2 diabetes, and other related renal and cardiovascular disease (Huang, 1999; Sprovieri and Sens, 2005; Alsafar et al., 2015). Besides, it is also a credible means to select patients who may benefit the most from therapy with angiotensin receptor blocker (ARB) (Ruggenenti et al., 2008). Apart from ACE, chymase, a serine protease that exists in mast cells, endothelial cells, stromal cells of the heart (Urata et al., 1993), and in vascular smooth muscle cells of the kidney (Huang et al., 2003), can also convert Ang I to Ang II (Urata et al., 1993; Takai et al., 2012). Chymase-hydrolyzed Ang II generation has emerged as a substitute for ACE in cardiac, vascular, and renal tissue, particularly in disease conditions (Bacani and Frishman, 2006; Miyazaki and Takai, 2006).

Recent research works have identified that ACE2, the first known human homolog ACE, is another carboxypeptidase and highly restricted to the heart, kidney, and testis in humans (Donoghue et al., 2000). As a counterbalance to ACE, ACE2 cleaves one aminoacid from Ang II, leading to the production of heptapeptide, angiotensin-(1–7)[Ang-(1–7)], which is generally considered to counter Ang II by activating Mas receptor (Mas) (Brand et al., 2002; Crackower et al., 2002; Ferrario and Chappell, 2004; Chamsi-Pasha et al., 2014; Romero et al., 2019). The Ang-(1–7)/Mas axis is downregulated with aging and may contribute to the aging-related susceptibility to neurodegeneration (Costa-Besada et al., 2018). Furthermore, ACE2 transforms Ang I into angiotensin-(1–9) [Ang-(1–9)] that, through the activation of angiotensin type 2 receptor (AT_2_R), may decrease blood pressure and protect the heart, blood vessels and possibly the kidney from adverse cardiovascular remodeling stimulated by hypertension or heart failure (Donoghue et al., 2000; Shariat-Madar and Schmaier, 2004; Ocaranza et al., 2014). Finally, these angiotensin family members can be further degraded into inactive peptide fragments, lowering the serum levels of endogenous vasodilators.

### Renin–angiotensin system receptors

Ang II binds with high affinity to two pharmacologically distinct G protein-coupled receptors, the AT_1_R and the AT_2_R (Karnik et al., 2015) (Figure 1). Studies have identified that two isoforms can be found in rats and mice, dubbed AT_1A_R and AT_1B_R with 94% of aminoacid sequence identity and pharmacologically indistinguishable (Sasaki et al., 1991; de Gasparo et al., 1995). The AT_1A_R, the closest murine homolog to the single human AT_1_R, is predominantly expressed in organs including the brain (Burson et al., 1994). While AT_1B_R is primarily expressed in the anterior pituitary gland and adrenal zona glomerulosa (Oliverio and Coffman, 2000). Most of the classical actions of Ang II are mediated by AT_1A_R, such as an increase in blood pressure, aldosterone synthesis and release from the adrenal zona glomerulosa, and stimulation of the sympathetic nervous system (Davisson et al., 2000). Overactivation of tissue Ang II leads to proliferation, fibrosis, inflammatory response, and oxidative stress, which appear to be associated with aging in certain tissues. AT_1B_R controls blood pressure when AT_1A_R is in shortage. Besides, Ang II can also bind to AT_2_R, which generally exerts opposing effects on those mediated by AT_1_R (Forrester et al., 2018). Ang II activating the AT_2_R induces vasodilation of arteries through nitric oxide (NO) and cGMP (cyclic guanosine monophosphate) stimulation and improves resistance artery remodeling, proliferation, and inflammation and decreases fibrosis and oxidative stress. These functions are expressed in the adrenal medulla, uterus, ovary, and distinct brain regions (Jones et al., 2008; Matavelli and Siragy, 2015; Sumners et al., 2015). AT_2_R also provides a cardio-protective effect against ischemia-reperfusion injury and acute myocardial infarction and protects the kidney from fibrosis and ischemic injury (Schulman and Raij, 2008). Moreover, reduction of senescence markers induced by Ang II was observed in AT_1A_R-inactivated mice, and disruption of AT_1A_R promoted the lifespan of mice compared with controls, possibly through attenuation of oxidative stress and upregulation of the pro-survival genes in the kidney, suggesting the role of AT_1A_R in senescence (Benigni et al., 2009). In addition, during the past 20 years, significant improvements have been made in understanding the mechanism and function of new members. Ang-(1–7) acts on the Mas, one that signals to mediate the antagonistic effect of Ang II, leading to vasodilation, NO release, anti-fibrosis, antiproliferation, and anti-inflammation. As with the binding of Ang-(1–7) to the Mas, alamandine also promotes the hypotensive effect through the production of NO with Mas-related G protein-coupled receptor D (MrgD) (Lautner et al., 2013). Alamandine [Ala^1^-Ang-(1–7)] is endogenously synthesized from Ang-(1–7), and therefore, its serum level can be regarded as amounting to that of Ang-(1–7) (Carey, 2013). Alamandine can also be formed from Ala^1^-Ang II (angiotensin A) by ACE2 owing to decarboxylation of the Asp residue (Lautner et al., 2013). Ang III binds to and activates the AT_1_R and AT_2_R, while Ang IV binds to the angiotensin type 4 receptor (AT_4_R), causing vasodilation in the vascular beds of the brain, heart, and kidney, improving memory and increasing renal cortical blood flow and natriuresis. Finally, prorenin and renin both act at the (pro)renin receptor (PRR) to induce specific effects. These effects may be related to aging, therefore, summarizing the relationship between the RAS and aging process may be of great significance for providing new strategies for humans to delay aging in the future.

## Angiotensin II, oxidative stress toxicity, and aging

**Figure 2:**
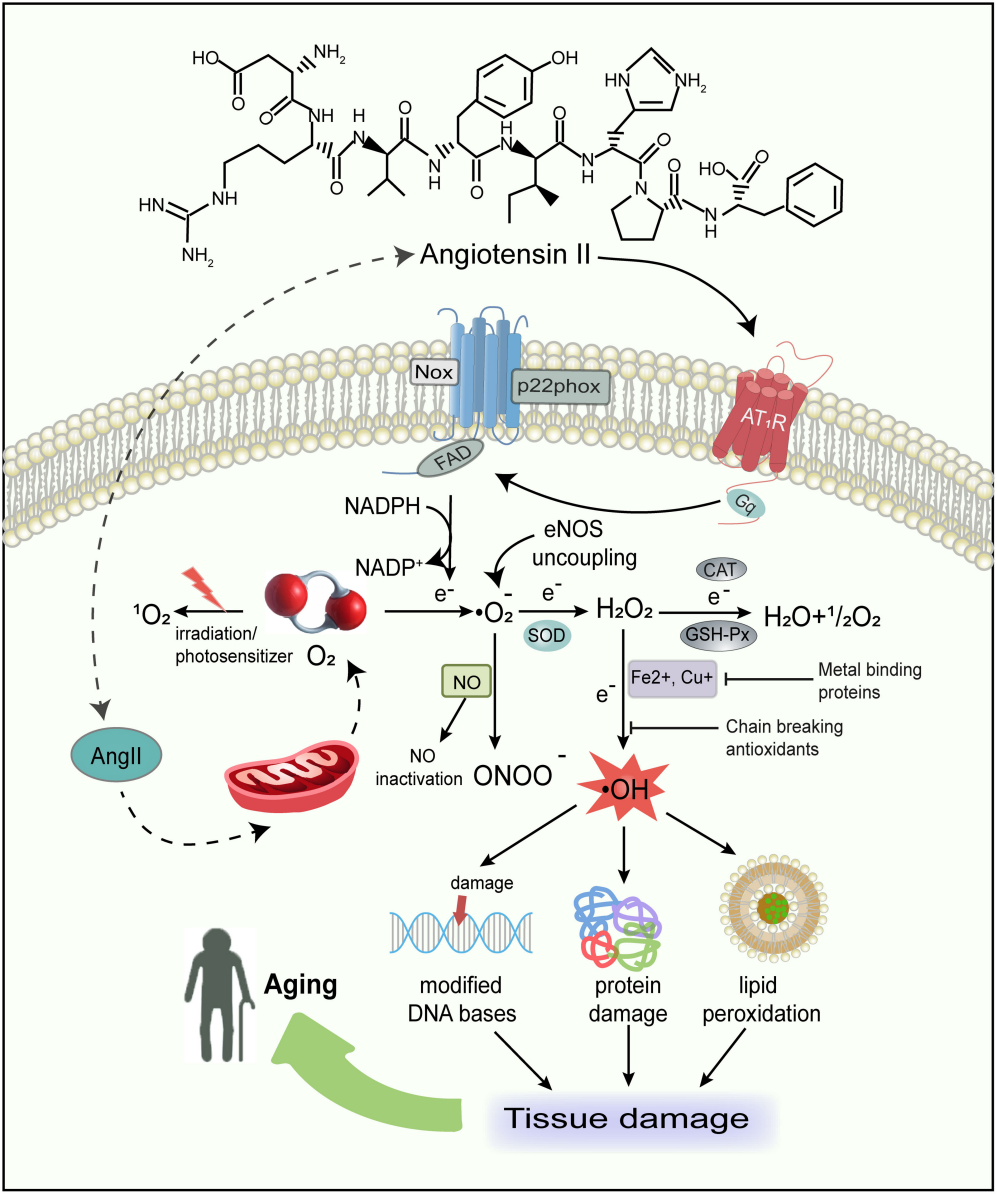
Schematic representation of major sources of free radicals in the body and the consequences of free radical damage. Ang II not only produces ROS by activating Nox and favoring eNOS uncoupling, but also has a direct action on mtROS production. The oxidative stress in turn can damage macromolecules, such as proteins, lipids, and DNA, therefore resulting in tissue damage and aging. ROS, reactive oxygen species; Nox, NAD(P)H oxidase; eNOS, endothelial NO synthase; mtROS, mitochondrial reactive oxygen species; O_2_, oxygen; ${}^{1}O_{2}$, singlet oxygen; ${\text{O}}_{2}^{•\text{–}}$, superoxide; SOD, superoxide dismutase; H_2_O_2_, hydrogen peroxide; H_2_O, water; OH^·^, hydroxyl radical; NO, nitric oxide; ONOO-, peroxynitrite; CAT, catalase; GSH-Px, glutathione peroxidase; NADPH, nicotinamide adenine dinucleotide phosphate.

As discussed above, in general, we have already laid out the functions of Ang II. It is a key player in the induction of the oxidative stress toxicity process and the activation of redox-sensitive signaling cascades (Zhang et al., 2007; de Cavanagh et al., 2011). An abundance of evidence has demonstrated that Ang II can activate NAD(P)H oxidase (Nox) (Griendling et al., 1994; Hanna et al., 2002; Kimura et al., 2005) to produce ROS *via* binding to AT_1_R and stimulate mitochondrial ROS (mtROS) production (Doughan et al., 2008; Dai et al., 2011), leading to oxidative stress damage (Figure 2). ROS can damage macromolecules, such as proteins, DNA, and lipids (Cross et al., 1987; Young and Woodside, 2001; Schieber and Chandel, 2014; Silva et al., 2018), resulting in the accumulation of genetic and biological activity alterations, acceleration of mutagenesis, and eventually cell death (Pagan et al., 2022). Therefore, accretion of Ang II-mediated damage to cellular macromolecules is deemed as an underlying driving force in the aging process.

In addition, Ang II also enhances NO generation in the cytoplasm of different cell types such as endothelial, vascular smooth muscle, tubular epithelial, and fibroblast cells. Since the interaction of NO with superoxide (${\text{O}}_{2}^{\cdot \text{–}}$) generates peroxynitrite (ONOO^−^) (Darley-Usmar et al., 1995; Fridovich, 1997; Pueyo et al., 1998), it can promote the production of both ROS and reactive nitrogen species (RNS), inhibit mitochondrial electron transport, and destroy DNA and cellular proteins (Radi et al., 1991). Furthermore, Ang II can promote endothelial NO synthase(eNOS) uncoupling, changing from NO to ${\text{O}}_{2}^{\cdot \text{–}}$ formation (Mollnau et al., 2002). Ang II-derived ROS, as significant intracellular second messengers, may activate many downstream signaling molecules such as mitogen-activated protein kinases (MAPK), tyrosine phosphatases, tyrosine kinases, and transcription factors (Madamanchi et al., 2005; Zhang et al., 2007). This suggests that ROS are highly related to regulating signal transduction pathways involved in cell growth, differentiation, apoptosis, and hypertrophy (Reckelhoff and Romero, 2003; Garrido and Griendling, 2009). We have previously reported that inhibition of MAPK induced by Ang II remarkably reduces senescence (Feresin et al., 2016), implying that Ang II-induced senescence is intricate and a combination of mechanisms may be involved.

### Generation of ROS and their biological activity

The reactive oxygen species are generated as intermediates in the cellular reduction–oxidation reaction of oxygen, converting oxygen to water (Figure 2). In the presence of a free electron (e^−^), the univalent reduction of oxygen yields ${\text{O}}_{2}^{\cdot \text{–}}$ anion, hydrogen peroxide (H_2_*O*_2_), and hydroxyl radical (OH^·^) (Table 1). Interestingly, oxygen, a substance indispensable for human life activities, has detrimental effects on humans under certain conditions (Pham-Huy et al., 2008). The majority of the potentially destructive effects of oxygen are attributed to the formation and activation of ROS, which are inclined to transmit oxygen to other biomolecules.

**Table 1:** ROS and the antioxidant defense mechanisms.

	**Abbreviation/formula**	**Characteristic**
**Pro-oxidants**		
Superoxide	${\text{O}}_{2}^{\cdot \text{–}}$	The earliest and most numerous free radicals
Hydrogen peroxide	H_2_*O*_2_	Lipid soluble; membrane permeable
Hydroxyl radical	OH^·^	Extremely high reactive; interacting with all types of molecules, resulting in membrane destruction, protein lipid peroxidation and DNA damage; no special scavenger
Singlet oxygen	^1^O_2_	The lowest electronically excited state of oxygen; a highly reactive molecule; generated by energy transfer (Mitra et al., 2019)
Nitric oxide	NO	Reacting with O${\text{O}}_{2}^{\cdot \text{–}}$ to give highly reactive oxidant ONOO^−^ and inactivate cytoprotective NO(Tsutsui et al., 2011)
Lipid peroxyl radical	LOOH	The product of many free radical reactions, usually on the cell membrane causing the cell membrane dysfunction
**Antioxidants**		
Antioxidant enzymes		Eliminating a large number of free radicals
• Superoxide dismutase	SOD	Removing excessive ${\text{O}}_{2}^{\cdot \text{–}}$free radicals in cells
• Catalase/Hydrogen Peroxidase	CAT	The first characterized antioxidant enzyme; greatest activity in liver and erythrocyte(Lobo et al., 2010)
• Glutathione peroxidases	GSH-Px	The highest concentration in liver; specific catalytic reduction of H_2_O_2_ by glutathione
Chain breaking antioxidants (Young and Woodside, 2001)		Directly scavenging free radicals; consumed during scavenging process
• Lipid phase	α-tocopherol/vitamin E	
	Ubiquinol	
	Carotenoids	
	Flavonoids	
• Aqueous phase	Ascorbate/vitamin C	
	Urate	
	Glutathione and other thiols	
Metal binding proteins		Sequestering iron and copper to prevent the formation of OH^·^
• Ferritin • Transferrin		
• Lactoferrin • Caeruloplasmin		

The reactive oxygen species play an important role in biological systems. Initially, ROS were considered as simple natural intermediate products involved in the molecular metabolism that can cause oxidative damage, various chronic diseases, and aging (Pham-Huy et al., 2008; Lobo et al., 2010; Rogov et al., 2021). However, with further research on free radicals in biology recently, they fulfill a more complicated and important physiological role than previously known, acting as vital intermediaries in cell signaling and homeostasis (Rogov et al., 2021).

The reactive oxygen species are highly diverse due to their respective dynamics and chemical properties, such as biological reactivity and the ability to cross membranes. The faster the elements in the ROS integrate with other molecules, the shorter the half-life. ROS can be charged or neutral, hydrophilic or hydrophobic, which dictates their capacities to cross biological membranes and move in aqueous or lipophilic environments. ${\text{O}}_{2}^{\cdot \text{–}}$, which is considered the primary ROS, has an unpaired electron in its molecular outermost orbital. In an aerobic chemical reaction, free radicals grab electrons from other substances to form a stable state because electrons must come in pairs. So, the unpaired electron of ${\text{O}}_{2}^{\cdot \text{–}}$ imparts high oxidation reactivity and renders ${\text{O}}_{2}^{\cdot \text{–}}$ unstable and short-lived. ${\text{O}}_{2}^{\cdot \text{–}}$ can work not only as an oxidizing agent, in which ${\text{O}}_{2}^{\cdot \text{–}}$ is reduced to H_2_*O*_2_ but also as a reducing agent, where ${\text{O}}_{2}^{\cdot \text{–}}$ provides its unpaired electron for NO to form ONOO^−^, leading to NO quenching and the resultant decrease in NO availability. The disproportionation of ${\text{O}}_{2}^{\cdot \text{–}}$ to H_2_*O*_2_ can be spontaneous or catalyzed by superoxide dismutase (SOD). There are three forms of SOD in mammalian tissues: cytoplasmic copper/zinc SOD (SOD1), mitochondrial manganese SOD (MnSOD/SOD2), and extracellular SOD (SOD3) (Griendling et al., 1994). NO is regarded as an important protective factor against cardiac-cerebrovascular disease since it modulates vascular dilator tone and local cell growth, downregulates the expression of adhesion proteins, and prevents platelet aggregation, thrombosis in blood vessels and smooth muscle proliferation (Tousoulis et al., 2012). Although H_2_*O*_2_, mainly produced by spontaneous ${\text{O}}_{2}^{\cdot \text{–}}$ dismutation, is not a free radical, it is usually a member of the general ROS. H_2_*O*_2_ may directly destroy enzymes or proteins involving active thiol groups under a weak oxidation process (Young and Woodside, 2001). Also, it is a lipid-soluble molecule that can cross cell membranes directly, while ${\text{O}}_{2}^{\cdot \text{–}}$can only go across cell membranes with the help of anion channels. Such a vital property allows H_2_*O*_2_ to diffuse over considerable distances from its site of production into other cells or cell compartments before decomposing to the highly reactive OH^·^ (Halliwell and Gutteridge, 1990; Touyz, 2003). In general, upon the situation of excess ${\text{O}}_{2}^{\cdot \text{–}}$ anions, OH^·^ is formed as a result of the decomposition of ${\text{O}}_{2}^{\cdot \text{–}}$ and H_2_*O*_2_ with transition metal (such as Fe^2+^ or Cu^2+^) catalyzed (Haber–Weiss reaction) (Koppenol, 2001). Although OH^·^ has a very short half-life *in vivo*, it is the strongest oxidant among the oxygen free radicals (Zheng et al., 2015) and the most dangerous ROS. It is considered the ultimate mediator of most free radical-induced toxic effects (Lloyd et al., 1997). Owing to the presence of an unpaired electron, OH^·^ could react at an extremely high rate with almost every type of biological molecule in living cells (del Río et al., 1992), especially with purines and pyrimidines in DNA, leading to cell death or mutation. All ROS discussed above exert tissue damage by inducing OH^·^ formation. There are no antioxidants to eradicate OH^·^ due to its extremely high reactivity.

### Antioxidant defense systems

Living organisms have evolved an extensive range of endogenous and exogenous antioxidant defense systems to protect cells against oxidative stress damage (Table 1). Free radicals-induced damage can be prevented by scavenging enzymes, as well as by other nonenzymatic antioxidants such as chain-breaking antioxidants, and transition metal-binding proteins (Figure 2). This can be realized by preventing the formation and scavenging of radicals or accelerating their decomposition (Finkel, 1998).

H_2_O_2_ is reduced to H_2_O and O_2_ by glutathione peroxidase (GSH-Px) and catalase. GSH-Px has higher affinity for H_2_O_2_ than catalase and is a vital antioxidant that not only prevents the formation of other more toxic free radicals, such as OH^·^, but also protects the cellular membrane from lipid peroxidation because glutathione contributes protons to the membrane lipids to keep them in a steady state. Furthermore, mice with GSH-Px overexpression were more resistant to myocardial oxidative stress and inhibited myocardial remodeling and failure in the heart of myocardial infarction, which might improve survival (Shiomi et al., 2004; Matsushima et al., 2006). Based on these lines of evidence, therapies aiming to interfere with oxidative stress may be a new avenue to prevent cardiac failure.

The rate and magnitude of oxidant formation and detoxification turn out to be balanced under normal physiological function. However, when the production of pro-oxidants overwhelms the body's antioxidative capacity, oxidative stress ensues (Lobo et al., 2010). As mentioned above, oxidative stress refers to the presence of excess free radicals in the nuclei and membranes of cells that attack lipids, nucleic acids, and proteins, triggering numerous chronic and degenerative illness diseases (Collin, 2019), such as PD (Trist et al., 2019) and Alzheimer disease (Tönnies and Trushina, 2017; Ionescu-Tucker and Cotman, 2021). Besides, it must be emphasized that cellular damage caused by oxidative stress is irreparable in that it induces irreversible macromolecular destruction, such as DNA or cellular membrane alteration or damage (Obrenovich et al., 2011). Hence, an external source of antioxidants should be applied prior to the development of oxidative stress.

### ROS and aging

The process of aging, a progressive functional decline and easier approach to death, has attracted boundless curiosity and deep exploration throughout human history. Although aging is a normal physiological process rather than a disease, it is recognized as the primary risk factor for all age-associated diseases (Rattan, 2014), such as cardiovascular disease, kidney failure, diabetes, cancer, and neurodegeneration, whose morbidity increases gradually along with aging (Brody and Grant, 2001; Lopez-Otin et al., 2013).

With the ever-increasing knowledge in molecular and cytology and continuous advances in technology, aging research has progressed unprecedentedly in recent years. Researchers surprisingly discovered that cancer and aging bear many similarities. They share mutual origins and are considered two different manifestations of the same potential process—the accumulation of cell damage. In addition, a lot of premature aging disorders result from the increased accumulation of DNA damage, such as Werner syndrome and Bloom syndrome (Burtner and Kennedy, 2010). In 2011, a landmark paper in the cancer field was published and enumerated 10 hallmarks of cancer (Hanahan and Weinberg, 2011); likewise, researchers have enumerated nine candidate hallmarks of aging, which is beneficial to conceptualizing the nature of age and its underlying mechanisms (Lopez-Otin et al., 2013).

The aging field first advanced a new concept in 1956, namely, the free radical theory of aging, with the publication of a significant article that proposed the basic chemical process of aging: the reaction between toxic ROS and cell cellular components (Harman, 1956). From then on, the free radical theory of aging was seriously questioned by many researchers. The identification of SOD in 1969 (McCord and Fridovich, 1969) and the following elucidation of cellular antioxidant defenses (Yu, 1994) gradually gave more credibility to the theory. There has been increasing evidence since then that the mechanism limiting longevity arises from ROS-induced damage (Harman, 1981; Barja, 2004). Subsequently, research in Britton Chance's lab found that oxygen in the mitochondrion is converted to H_2_O_2_ (Chance et al., 1979), which prompted Jaime Miquel to extend the free radical theory of aging and formulate in the early 1980s, the mitochondrial free radical theory of aging: oxidative stress damage to mitochondrial DNA (mtDNA) may result in mutation and replication arrest, and mitochondrial function impairment during aging (Miquel et al., 1980; Fleming et al., 1982). It became evident that the proposed theory initiated to link the accumulation of ROS with aging. ROS destroy the structure of cell membrane and protein function and mutate or delete genes through molecular peroxidation, which in turn critically influences the life span. Although substantial evidence that oxidative stress toxicity increases in aging, the theory is still seriously doubted by some scientists (Lapointe and Hekimi, 2010; Gladyshev, 2014). The question of the specific mechanism of how ROS act on aging remains unresolved.

Surprisingly, low ROS levels were likely to extend longevity in *C. elegans* (Palikaras et al., 2015). Similarly unexpected, elevating the levels of ROS in Drosophila *via* respiratory complex I could act as a signal to delay aging (Scialò et al., 2016), and finally, mice with overexpression of antioxidants did not present a prolonged lifespan (Pérez et al., 2009). These findings appear to contradict the destructive effects of ROS. In fact, in parallel studies of the detrimental effects of ROS, the area of the intercellular signal has experienced a rapid advance. Convincing evidence has accumulated that low levels of ROS may function as signaling molecules, improve systemic defense mechanisms by inducing an adaptive response (referred to as mitohormesis) to fight against cellular stress and damage with age, and eventually extend lifespan (Ristow and Schmeisser, 2014). Whereas, excessive ROS can defeat their original homeostatic goal and cause increased age-related damage and shortened lifespan (Hekimi et al., 2011; Gonzalez-Freire et al., 2015). Indeed, activating slight mitohormesis in Drosophila muscle significantly extends lifespan through systemically antagonizing insulin signaling, facilitating mitophagy, and regulating the mitochondrial unfolded protein response (Owusu-Ansah et al., 2013).

### Ang II, cell senescence, and aging

Under physiological conditions, Ang II-regulated ROS and RNS production and the subsequent promotion of oxidative stress are tightly controlled. On the contrary, under pathological conditions associated with the RAS overactivation, such as hypertension, atherosclerosis, myocardial infarction (Steinberg et al., 1989; Dhalla et al., 1996), diabetes (Rincon-Choles et al., 2002), and aging (Baylis et al., 1997; Thompson et al., 2000; Wang et al., 2003), the dysregulation of Ang II-dependent ROS generation may become a critical contributor to cell oxidation and connective tissue damage. Previous experiments showed that cell senescence was faster after exposure of human glomerular mesangial cells to Ang II (Feng et al., 2011). Recent research work has demonstrated that the pervasiveness of senescent vascular smooth muscle cells increases with age and can be accelerated by multiple Ang II-induced stress responses, including oxidative lesions, inflammation, and mitochondria dysfunction (Okuno et al., 2020).

In 1956, Harman proposed that ROS are the most significant molecular species involved in the aging process (Harman, 1956). Based on his theory, aging and age-associated degenerative diseases are attributed primarily to the deleterious side attacks of ROS. Hence, Ang II likely takes part in the aging process considering its capacity for mediating the generation and release of ROS. The upregulated activities of the RAS have been observed in aging mice kidneys. Here, AT_1_R was activated that accelerated ROS generation, as well as tubular senescence and renal fibrosis in D-galactose, establishing the aging model. These effects were blocked by treatment with AT_1_R blocker losartan (Miao et al., 2019). Recent advance in the field of cardiovascular diseases indicates that overactivation of Ang II is seriously harmful to cardiac function, including exacerbated fibrosis, oxidative lesion, and hypertrophy (Zablocki and Sadoshima, 2013; Hamilton et al., 2016). The later data showed that, in contrast to Ang II alone, mice administered with the combination of Ang II and L-NG-nitroarginine methyl ester (inhibition of NO synthesis) exerted a pernicious impact on mitochondria and exacerbated oxidative damage, leading to more deleterious heart failure (Hamilton et al., 2016). This result might present new therapeutic opportunities that modify mitochondrial damage and improve the redox state to prevent the incidence of heart failure. In animal models of PD, excessive brain AT_1_R stimulation enhanced oxidative stress and injured dopaminergic cells (Dominguez-Meijide et al., 2014), while ARB could enter the brain and decrease excessive brain inflammation and neuronal injury (Villapol and Saavedra, 2015). Clinical studies showed that ARB improved cognitive loss after stroke and aging. Since in brain cells, the intracellular RAS counteracts the oxidative stress induced by the extracellular/paracrine Ang II, ARB acting only on the extracellular or paracrine RAS may give cells better protection (Villar-Cheda et al., 2017). Overall, blocking the RAS using different antagonists, such as valsartan, candesartan, and telmisartan, as a possible strategy to reduce aging, would be advisable and would provide a more translational vision (Basso et al., 2005; Villapol and Saavedra, 2015).

Moreover, it has been shown that increasing ROS levels by exposing cells to exogenous H_2_O_2_ or enhancing the partial pressure of oxygen could induce human fibroblasts senescence with irreversible cell cycle arrest (von Zglinicki et al., 1995; Chen et al., 1998). Conversely, antioxidants, such as apocynin, kaempferol, and polyphenol extracts, markedly attenuated Ang II-induced oxidative damage and cellular senescence (Wang et al., 2013; Feresin et al., 2016; Du et al., 2019).

## Ang II and mitochondrial dysfunction

### Mitochondria as a source of ROS

Mitochondria were described in the early 1890s as ubiquitous intracellular structures (Ernster and Schatz, 1981). Over the past century, with technological advances, several research studies elaborated on many comprehensive molecular details of mitochondria, including mitochondrial origin, construction, gene, metabolism, and signaling pathways (Son and Lee, 2021).

**Figure 3:**
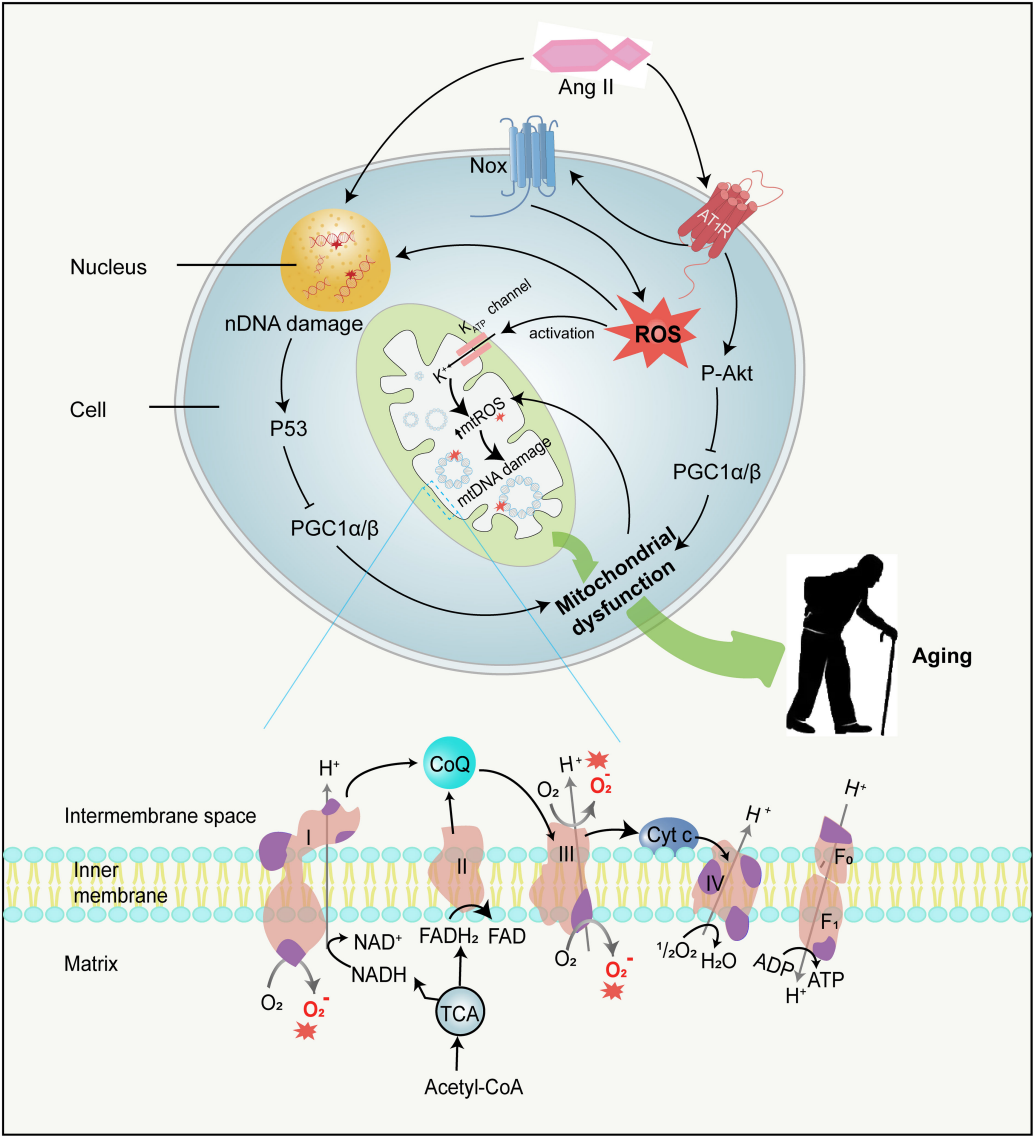
Ang II and mitochondrial dysfunction. Ang II can not only damage mtDNA by stimulating mtROS production in several ways but also damage nDNA directly. MtDNA mutations directly accelerate mitochondrial dysfunction, while nDNA damages result in mitochondrial dysfunction by activating P53 and subsequently inhibiting PGC-1α*andPGC*−1β, therefore contributing to aging. Besides, Ang II binding to AT_1_R activates Akt and in turn phosphorylates PGC-1α/β, leading to mitochondrial dysfunction. The mitochondrial ETC is composed of four respiratory complexes (1-IV) and ATP synthase(F_0 − 1_), which are all made up of proteins encoded by both mitochondrial (purple) and nuclear (pink) genes. MtROS (red asterisk) are primarily generated from complexes I and III in the ETC. mtDNA, mitochondrial DNA; nDNA, nuclear DNA; PGC-1α and PGC-1β, peroxisome proliferator-activated receptor gamma coactivator 1-alpha and -beta; ETC, electron transfer chain; ATP, adenosine triphosphate; Cyt c, cytochrome c; ADP, adenosine diphosphate; NADH, nicotinamide adenine dinucleotide; FAD, flavin adenine dinucleotide; FADH_2_, flavine adenine dinucleotide reduced; CoQ, Coenzyme Q; TCA, tricarboxylic acid cycle.

Mitochondria were once viewed as critical subcellular organelles dedicated to the generation of energy to keep the endergonic biochemical processes of cell life through the tricarboxylic acid cycle (TCA cycle). Now, they are regarded as remarkably multifunctional double membrane organelles that function as a crucial regulatory station for executing and coordinating essential cellular metabolism, ranging from calcium homeostasis (Nicholls, 2005), immune response (Pinegin et al., 2018), cell apoptosis (Brookes et al., 2000), tissue oxygen gradients (Thomas et al., 2001; Collins et al., 2012) to intracellular signaling (Cadenas, 2004). In the process of oxidative phosphorylation, mitochondria transfer electrons to oxygen through the electron transfer chain (ETC), producing the vast majority of cellular adenosine triphosphate (ATP). Although the mitochondrial ETC is a very efficient system, such an energetic process does have its downsides, and the ETC has been demonstrated to be somewhat “leaky”. During this process called “electron leak”, the “leaky” mitochondrial electron can spontaneously side react with some components of the respiratory chain to generate ROS, mainly from complexes I and III of the ETC (Cadenas and Davies, 2000; St-Pierre et al., 2002; Barja, 2014; Zhang et al., 2018) (Figure 3). For example, while the ubiquinone in the ETC cycle, from the states of quinone to semiquinone and to quinol, electrons tend to be passed directly to the oxygen molecule rather than to the next electron carrier in the respiratory chain. Furthermore, a few iron–sulfur clusters also generate toxic ${\text{O}}_{2}^{\cdot \text{–}}$ with oxygen during the respiratory chain (Cadenas and Davies, 2000). Apart from these ETC reactions in the mitochondrial inner membrane, another source of ROS is monoamine oxidases in the mitochondrial outer membrane which is not associated with the respiratory chain. The oxidative deamination of biogenic amines catalyzed by monoamine oxidases can directly reduce two-electron of ${\text{O}}_{2}^{\cdot \text{–}}$ to H_2_*O*_2_ (Hauptmann et al., 1996), which contributes to the modulation of ROS signaling and the maintenance of oxidative homeostasis within the mitochondrial matrix and cytosol. According to a putative vicious cycle, ROS derived from mitochondria can potentially feed back to the mitochondria forming more mtROS (Zorov et al., 2014). Therefore, mitochondria are commonly regarded as the major source of intracellular ROS under physiological conditions.

### Oxidative damage in mitochondrial and nuclear DNA

Mitochondria consume more than 90% of cellular oxygen to generate energy to meet the needs of cellular activities. While most oxygen is converted into water, approximately 2% forms ROS (Boveris and Chance, 1973). However, mtROS, especially OH^·^, can react with almost every type of cell macromolecule and destroy their functions, thereby compromising mitochondrial integrity and function. Although proteins and phospholipids are turned over without perpetual damage, unrepaired ROS-induced nucleic acid damage can persistently exist and accrete over time. It is worth noting that both the mtDNA and nuclear DNA (nDNA) can be damaged by mtROS. In a previous study (Van Remmen et al., 2003), the MnSOD activity was reduced by about 50%, and the level of endogenously generated ROS was significantly increased in tissues of mice heterozygous for the SOD2 gene compared with wild-type mice. These heterozygous mice were also notably shown to exhibit elevated levels of 8-oxo-2-deoxyguanosine (8-oxoG) in both mtDNA and nDNA, which is both one of the most abundant mutations brought by oxidative conversion of guanosine and an important marker of oxidative stress. The increased 8-oxoG denoted a rising incidence of mtDNA and nDNA damage as well as a high rate of tumor formation in heterozygous SOD2 mice. Based on this finding, a conjecture that mitochondrial oxidative injury might be a considerable element of entire genomic instability started to attract the attention of researchers and was gradually embraced by them. The hypothesis is also supported by an experiment in irradiated human fibroblasts, in which exogenous ROS production leads to mitochondrial dysfunction, nDNA damage acceleration, and senescent phenotype through initiating signaling and forming a positive feedback loop (Passos et al., 2010). Similarly, mitochondrial oxidants can destroy nDNA (Balaban et al., 2005; Singhapol et al., 2013; Douiev et al., 2018), probably owing to the facts that several mtROS enhance oxidative lesions regarding the mitochondrial, cytosolic, and nuclear compartments (Van Remmen and Richardson, 2001), and that some ROS, e.g., H_2_O_2_, can spontaneously cross the mitochondrial membrane resulting in nuclear damage (Van Remmen et al., 2003). Although nDNA possesses repair mechanisms, the cell activates a series of signal transduction pathways if the damage is too large to repair (Figure 3). Damaged nDNA is further capable of promoting the phosphorylation of p53 (Balaban et al., 2005; Sahin and DePinho, 2012), which subsequently couples with the promoters of peroxisome proliferator-activated receptor gamma coactivator 1-alpha and –beta (PGC-1α and PGC-1β) and suppresses their expression (Sahin et al., 2011; Sahin and DePinho, 2012). PGC-1α and PGC-1β coordinate sophisticated metabolic responses including mitochondriogenesis, antioxidant defenses enhancement, and aliphatic acid oxidation improvement (Fernandez-Marcos and Auwerx, 2011; Feresin et al., 2016) and are regarded as the “master regulators” of mitochondria. The repression of both PGC-1α and PGC-1β finally affects entire mitochondria biogenesis, function, as well as homeostasis, thus leading to deficient ATP production and ROS enhancement (Scarpulla, 2011; Sahin and DePinho, 2012). In addition to mitochondrial dysfunction, decreased PGC-1α also reduces telomerase activity and increases DNA damage, resulting in telomere attrition and replicative senescence (Xiong et al., 2015). Unfortunately, the influence of p53 on mitochondriogenesis and subsequently caused cell pathophysiological mechanism is not overall clear. Additional research is needed to elucidate the exact mechanism of DNA damage in mitochondrial ETC dysfunction and the corresponding measures of prevention.

However, mtDNA is commonly considered to be more sensitive to oxygen radical attack, which can be ascribed to multiple factors. mtDNA is in the proximity of the reactive species generation site and exposed to a high concentration of reducing metabolites environment (Cadenas and Davies, 2000; Son and Lee, 2021); mtDNA lacks protective histones and has a relatively limited repair mechanism compared to nDNA (Linnane et al., 1989; Ames et al., 1995). As a corollary, the amount of oxidized bases in mtDNA is 10–20 times higher than that in nDNA(Richter et al., 1988; Adachi et al., 1995).

Importantly, over 900 proteins exist in mitochondria that are encoded by both nuclear and mitochondrial genomes. Although the majority of mitochondrial proteins are encoded by the nuclear genes, the mitochondrial genome is indispensable in the process of mitochondriogenesis, which is responsible for offering the templates for 13 essential proteins of ETC, and mitochondrial tRNAs and rRNAs transcription (Gomez-Cabrera et al., 2012; Lauri et al., 2014). Unlike wild-type mtDNA, mutated mtDNA is easier and faster to replicate, and in turn, contributes to its clonal expansion. If the rate of mutated mtDNA increases to 60%, insufficient respiration occurs in the cell and initiates a vicious spiral of progressively increasing mtDNA mutations (Lauri et al., 2014; Chinnery, 2015). Collectively, the extent of mtDNA base oxidation and deletion is increased with the H_2_O_2_ generated by inner (ETC) or outer (monoamine oxidase) membrane activities. Thus, mitochondria are, *per se*, targets of ROS/RNS-mediated damage, leading to the accumulation of mtDNA mutations, mitochondrial dysfunction, and therefore several diseases, such as cancer, mitochondrial diseases, aging, and age-related diseases (Chinnery, 2015; Yan et al., 2019; Fontana and Gahlon, 2020).

### Oxidative damage to MtDNA as an aging driving force

As cells and organisms age, proteasome activity descends, autophagy is inactivated, and mitochondrial function becomes perturbed, which emerges with the diminishing efficacy of the respiratory chain and ATP generation as well as the growing electron leakage and oxidative stress (Green et al., 2011; Theurey and Pizzo, 2018). Besides, it has been reported that in various organisms, oxidative injury to mitochondrial lipids, DNA, and proteins are highly augmented during the aging in some tissues (Van Remmen and Richardson, 2001). Interestingly, the mitochondria-targeted antioxidant mitoquinone can improve mitochondrial mass and diminish renal cell senescence and renal fibrosis in constituted aging models (Miao et al., 2019). Mutator mice overexpress catalase specifically in mitochondria, which decreases ROS-inflicted destruction and substantially ameliorates cardiac aging, evidenced by the important role of maintaining mitochondrial function in longevity (Dai et al., 2009, 2010). Oxidative stress is known to be the main reason for physiologic function decline in the aging process. Since mitochondria are a major site for ROS production, the relationship between mitochondrial function impairment and aging has long been attracting attention and suspicion. Anatomizing their relationship remains a key challenge in carrying out research work on aging.

Later, data greatly supports the importance of mtDNA damage in aging and age-related diseases. The first clear evidence comes from the observation and authentication of multisystem diseases caused by mtDNA mutations that partially phenocopy aging (Wallace, 2005). More convincing evidence that mtDNA point mutations or defects are a driving force behind the aging phenomenon derives from the creation of transgenic mice expressing a proofreading deficient mitochondrial DNA polymerase γ. These mutator mice show a higher rate than wild-type mice in mitochondrial mutation accumulation and an obvious early aging phenotype since the 25th week, such as osteoporosis progress, hair loss, and reduced fertility. Besides, a mosaic pattern related to cytochrome c oxidase (complex IV) deficiency, mitochondrial abnormalities, and enlargement is also observed in these transgenic mice. Finally, their life span averages 48 weeks (Trifunovic et al., 2004; Kujoth et al., 2005; Vermulst et al., 2008). Overall, the premature aging phenotype and short lifespan are associated with the accumulation of mtDNA mutations.

The continuous production of ROS and RNS by mitochondria provides evidence of the mitochondrial aging free radical theory, as an extension of the more general aging free radical theory (Miquel, 1998; Barja, 2014; Ziada et al., 2020). Based on the updated free radical theory of aging, oxidation of mitochondrial components destroys mitochondrial function primarily through mtROS, which in turn amplifies mtROS production and oxidizes macromolecule, resulting in deteriorating cellular and organ function. Afterward, evidence highly in favor of the mitochondrial version of the free radical aging theory is presented in a review (Barja, 2004) that generalizes two known features associating aging with oxidative stress damage by a comparative analysis of animals with different lifespans. Barja discovered that mtROS production was positively correlated with mtDNA destruction while negatively related to maximum longevity. Also, the unsaturated level of tissue fatty acid was linked inversely with maximum lifespan. Despite substantial evidence that mtDNA mutations are enhanced during aging, some recent findings have provided grounds for questioning the correlation between oxidative damage and mtDNA mutations. For instance, an experiment observed no increase in mtDNA transversion mutations caused by oxidative lesions, but an increase in mtDNA transition mutations within aging (Trifunovic et al., 2004; Kennedy et al., 2013). Therefore, it is inferred that rather than the direct function of oxidative stress, ROS may contribute to mtDNA mutations *via* altering mitochondrial polymerase γ, reducing its fidelity, and indirectly increasing somatic transition mutations (Kauppila et al., 2017; Ziada et al., 2020). ROS may as well affect mitochondrial biogenesis *via* signaling molecule pathway and, in turn, promote replication of existing mtDNA mutations (Ziada et al., 2020).

### Link between Ang II and mitochondrial dysfunction

Recently, it has become increasingly apparent that the generation of ROS is connected with the reciprocal interaction between the RAS and mitochondria, the latter of which are major ROS sources. Several studies support the direct interactions between Ang II and nuclear and mitochondrial components. First, ^125^I-labeled Ang II was tested in cellular mitochondria and nuclei of rodents (Robertson and Khairallah, 1971; Sirett et al., 1977). Second, renin, angiotensinogen, and ACE were also found in intramitochondrial dense bodies of rat adrenal glands (Peters et al., 1996). In human and mouse cells, AT_1_R and AT_2_R were detected within the inner membrane of mitochondria and interacted with Ang II (Abadir et al., 2011); moreover, AT_1_R was also detected in cell nuclei (Booz et al., 1992; Eggena et al., 1993). Finally, studies showed that angiotensinogen was internalized by renal proximal tubule cells and trafficked to the nucleus and mitochondria. Studies also showed that the precursor was taken up by isolated mitochondria as well. Besides, Pendergrass et al. (2009) reported that AT_1_R sites in renal cells were coupled to ROS generation likely through NOX4.

Ang II is discovered to stimulate mtROS production (Doughan et al., 2008; Dai et al., 2011) (Figure 3), which thereby represses the mitochondrial respiratory chain and decreases the energy of ATP. A study has demonstrated that activation of the Ang system by the AT_2_R in mitochondria is accompanied by mitochondrial NO generation and is involved in the regulation of mitochondrial respiration (Abadir et al., 2011). Additionally, Ang II binding to AT_1_R could phosphorylate PGC-1α by activating Akt, which decreases the binding of transcription factor forkhead box O 1 (FoxO1) to catalase and Sirt1 promoters, reducing the expression of these target genes. Sirt1 deficiency further increases PGC-1α and FoxO1 acetylation, forming a negative feedback loop where mitochondrial dysfunction and cell senescence are accelerated (Xiong et al., 2010; Feresin et al., 2016; Salazar, 2018). Concomitantly, Ang II could also modulate the PKC(protein kinase C)/AMPK (adenosine monophosphate-activated protein kinase)/ULK1(unc-51 like autophagy activating kinase 1) axis and inhibit the autophagic flux to exacerbate lipid deposition and mitochondrial dysfunction (He et al., 2019).

It is necessary to elucidate the mechanism of how Ang II promotes the generation of mtROS. Some research has proved that ROS produced through Nox could facilitate the further generation of ROS *via* other channels. For instance, ${\text{O}}_{2}^{\cdot \text{–}}$ originated from Nox might uncouple NO synthase to form ${\text{O}}_{2}^{\cdot \text{–}}$ by oxidizing and degrading tetrahydyrobiopterin (BH4) (Laursen et al., 2001; Landmesser et al., 2003). Likewise, H_2_*O*_2_ can promote further ROS production *via* stimulating dehydrogenase to form xanthine oxidase (McNally et al., 2005). So, Ang II-mediated mtROS formation may also be triggered by Nox-originated ROS. The review of Zhang et al. (2007) supports this hypothesis and clarifies this mechanism in detail. Ang II activates Nox to increase cytosolic ROS levels, which in turn induces the opening of mitochondrial ATP-dependent potassium channels (mitoK_ATP_) in the inner mitochondrial membrane, depolarizes mitochondrial membrane potential, and results in a burst of mtROS. Subsequently, mtROS are required for activation of downstream signaling cascades and autophagy (Yu et al., 2014), thus promoting cell apoptosis and senescence. Another piece of evidence that relates Ang II to mtROS presented by the experiment indicates that mitochondrial p66^Shc^ deletion could protect from Ang II–inflicted myocardial hypertrophy (Graiani et al., 2005). Partly situated in the intermembrane space of mitochondria, p66^Shc^ was thought to subtract electrons from cytochrome c and transfer them to oxygen to produce mtROS (de Cavanagh et al., 2015). Another fact worth mentioning is that the mtROS production stimulated by Ang II is related to vascular cell adhesion molecule-1. Ang II prompts the release of vascular cell adhesion molecule-1, which, like Ang II, can activate Nox and the consequent mtROS generation (Pérez et al., 2003; De Giusti et al., 2008). Ang II-mediated endothelial dysfunction has been reported in a variety of cardiovascular diseases (Dimmeler et al., 1997). Ang II, through attracting excessive expression of ROS in both cytoplasm and mitochondria, is involved in DNA damage, mitochondrial dysfunction, and inflammation, ultimately causing endothelial apoptosis (Caporali and Emanueli, 2011; Mendell and Olson, 2012; Zhang Y. et al., 2016; Li et al., 2019; Miyao et al., 2020). Later, it was shown that temporary Ang II stimulation promoted cell viability, including the ability of cell proliferation, migration, and angiogenesis (Li et al., 2019). While Ang II has long been considered a key regulator of endothelial apoptosis, the underlying mechanisms remain to be explored.

A study (Abadir et al., 2011) showed that mitochondrial AT_1_R increased and AT_2_R declined in mice kidneys along with age, and these consequences were combated by losartan. This may offer a novel way to decrease mitochondrial damage and chronic disease burden with aging. Moreover, apart from RAS activity enhancement, the quality and function of mitochondria were also destroyed with time in aging mice (Miao et al., 2019). This study has demonstrated that RAS signaling plays a primary role in the relationship between RAS-mediated age-associated renal fibrosis and mitochondrial dysfunction, and that inhibition of the signaling can efficiently attenuate renal fibrosis. Irbesartan, a typical ARB, considerably reversed mitochondrial dysfunction induced by Ang II by improving ATP production and mitochondrial potential and decreasing the amounts of ROS(He et al., 2019). In the future, thus, inhibition of RAS activity may be a feasible and practical therapy to ameliorate the progress of mitochondrial damage.

To sum up, an increasing number of studies show that mtROS are involved in the deleterious effects of Ang II, which are probably mediated by the activation of AT_1_R or direct interaction between Ang II and mitochondrial or nuclear components. Moreover, the existence of the RAS in human mitochondria contributes to understanding the interaction of mitochondria with aging and exposes underlying therapeutic targets to attenuate mitochondrial dysfunction and chronic disease during aging.

## Ang II and telomere attrition

### Telomere, telomerase, and aging

The intimate relationship between telomere attrition and cell senescence or aging has long been widely accepted (Allsopp et al., 1992; Kruk et al., 1995). With a growing understanding of the diverse molecular mechanisms of aging, the telomere is generally considered an instigator and amplifier of molecular loopbacks which pushes the aging process and degenerative diseases (Harley et al., 1990; Hayflick, 2000; Chakravarti et al., 2021) discovered a phenomenon, known as the Hayflick limit, that human fibroblasts stopped dividing after 40 to 60 times. Furthermore, telomere shortening occurred in parallel with the replicative lifespan of human fibroblasts, confirming telomere attrition could drive replicative senescence.

**Figure 4:**
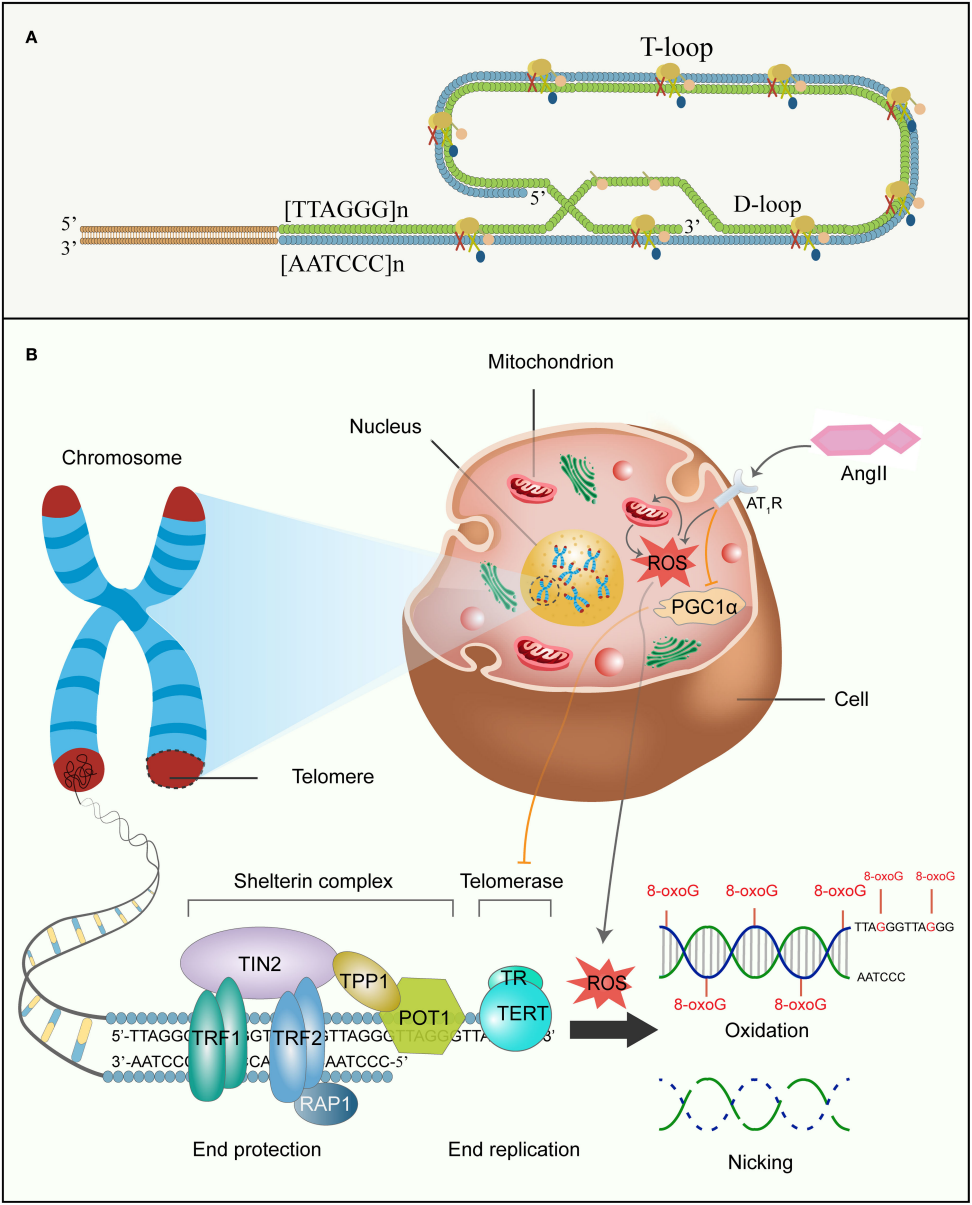
Ang II and telomere attrition. **(A)** Shelterin complex, structure of the D-loop and T-loop at the end of telomere. **(B)** Ang II, telomeres, and senescence. Telomeres are at the ends of linear chromosomes and combine with shelterin multiprotein complexes to escape from DDR. Shelterin complex comprises six diverse protein subunits: TRF1, TRF2, POT1, TIN2, TPP1, and RAP1, and are involved in chromosome end protection, while telomerase is made up of TERT and TR to function as end replication. However, the G-rich chain of telomeric DNA is sensitive to ROS induced by Ang II, and further accumulates 8-oxoG damages and forges high frequency of single-strand breakage in the DNA backbone. Concomitantly, Ang II also reduces telomerase activity by decreasing PGC-1α function. All these eventually accelerate replicative senescence. DDR, DNA damage response; TRF1 and TRF2, telomeric-repeat-binding factor 1 and 2; POT1, protection of telomeres 1; TIN2, TRF1-interacting nuclear protein 2; TPP1, TIN2-interacting protein; RAP1, repressor and activator protein 1; TERT, telomerase reverse transcriptase; TR, telomerase RNA; 8-oxoG, 8-oxo-2-deoxyguanosine.

Telomeres are tandem repetitive 5′-TTAGGG-3′ nucleotide sequences at chromosome ends that contain proximal double-stranded and distal single-stranded regions. Telomeres combination with protegerin complex preserves the terminals from being regarded as DNA damage and therefore maintains chromosomal integrity and genomic stability (Figure 4A) (Chan and Blackburn, 2004). The Telomeric G-tail of single-stranded nucleotide is tucked into the duplex DNA to form a lariat-like structure named T loop. Then, the T loop forms a triple DNA strand secondary structure called a d-loop (Griffith et al., 1999; Doksani et al., 2013; Smith et al., 2020). Although telomeres are constitutionally unstable, vulnerable parts, they become stabilized by interacting with a distinctive so-called shelterin. Shelterin, a multiprotein complex, can disguise chromosome ends from DNA damage response (DDR) signals, prevent DNA repair systems from fusing ends by recombination or classical/alternative non-homologous end joining, and regulate telomerase access and activity at the end (Sfeir et al., 2009; Sfeir and de Lange, 2012). It comprises three protein subunits, namely telomeric-repeat-binding factor 1 and 2 (TRF1 and TRF2) and protection of telomeres 1 (POT1) that directly bind to DNA and three other subunits, namely TRF1-interacting nuclear protein 2 (TIN2), TIN2-interacting protein (TPP1), and repressor and activator protein 1 (RAP1), which regulate the interaction between the components (Figure 4B) (Martínez and Blasco, 2011; Shay and Wright, 2019). While TRF1 and TRF2 as homodimers both recognize the double-stranded DNA and effciently promote telomere replication, POT1 binds with high specificity and affinity to the single-stranded 3′ overhang (Schmutz and de Lange, 2016; Erdel et al., 2017). TIN2 bridges TRF1 and TRF2 by simultaneously interacting with the two proteins *via* independent domains and recruits the TPP1–POT1 complex (Hu et al., 2017; Kalathiya et al., 2018). RAP1 binds directly to TRF2 in order to localize at the telomere (Martinez et al., 2010). Thus, the higher-order structure of telomeres solves the end protection problem by covering telomeric DNA. Correspondingly, mutations or losses in the aforementioned constituents can impair the telomere–shelterin complex and the binding of telomerase-telomeric ends, resulting in telomere attrition and premature senescence (van Steensel et al., 1998).

Incomplete replication at chromosomal ends gives rise to gradual telomere attrition during every consecutive cell mitosis (O'Sullivan and Karlseder, 2010). On account of the unidirectional activity of replicative DNA polymerase, it lacks the ability to fully replicate the ends of linear DNA molecules. Consequently, each division would result in a progressive reduction in the chromosomal telomere length because of removing termini RNA primer for lagging-strand synthesis; finally, this shortens the coding sites of the DNA. To avoid this, when the length of somatic cell telomere is reduced to a critical level or is damaged, the region is considered to have double-stranded DNA breaks and activate P53 and P21, causing cell growth arrest and ultimately triggering replicative senescence (Olovnikov, 1973; d'Adda di Fagagna et al., 2003; Oganesian and Karlseder, 2009; Aunan et al., 2016). To a certain degree, some cells, including germ cells (Lee et al., 1998), cancer cells (Singhapol et al., 2013), hematopoietic cells (Colla et al., 2015), and embryonic stem cells of the skin(Lee et al., 1998) and gastrointestinal (Schepers et al., 2011), mitigate shortening through expressing holoenzyme telomerase, which repetitively adds successive 5′-TTAGGG-3′ to maintain telomere homeostasis and overcome the end replication problem (Barnes et al., 2019). Unfortunately, most human somatic cells are short of telomerase, and the critically shortened telomere is incapable of adequate shelterin binding parts, which leads to the chromosomal terminal being exposed to DDR and then the telomere being falsely considered as DNA breaks. The core telomerase is made up of a catalytic protein subunit termed telomerase reverse transcriptase (TERT), as well as a non-coding RNA subunit, termed telomerase RNA (TR) that involves the RNA template for telomere synthesis (Blackburn et al., 2006; Singhapol et al., 2013). Bodnar et al. have found that ectopic expression of TERT in normal human cells renders the cells with the ability to replicate immortally rather than induce malignant conversion (Bodnar et al., 1998). Importantly, numerous data have discovered a positive association of telomere length with age-linked diseases (Blasco, 2005) such as atherosclerosis (Hunt et al., 2015), atrial fibrillation (Carlquist et al., 2016) and osteoarthritis (Kuszel et al., 2015). Moreover, studies on long-lived families (Honig et al., 2015) as well as Ashkenazi centenarians and their offspring (Atzmon et al., 2010) also identified such association and uncovered that telomere length shortened with age and that those with longer telomeres had better overall health.

Further evidence in favor of the importance of telomeres in the human lifespan stems from the study of telomere maintenance-related gene mutations or losses in humans. For example, patients with dyskeratosis congenita carried gene TERT and TERC mutations, which resulted in shortened telomeres and reduced lifespan (Kirwan and Dokal, 2009). Other evidence comes from observation of patients with premature aging disorders, including Werner syndrome and ataxia telangiectasia (Martin, 2005). Interestingly, mutations in TERC and TERT alike had relevance to organ-restricted conditions, such as liver cirrhosis, idiopathic pulmonary fibrosis, and bone marrow failure syndromes (Armanios and Blackburn, 2012). Concomitantly, studies reported aplastic anemia and dyskeratosis congenita possessed mutations in the shelterin protein complex, particularly of TINF2 (Savage et al., 2008; Walne et al., 2008). Those diseases are featured decreased tissue regeneration and expedited aging (Armanios and Blackburn, 2012). Finally, an experiment proved that reactivation of the telomerase gene could reverse premature aging in telomerase-lack mice (Jaskelioff et al., 2011).

Aside from functioning as a maintenance telomere, however, telomerase, TERT, in particular, serves many non-telomere-linked functions including regulation of growth factors, gene expression, and cell multiplication (Smith et al., 2003; Li et al., 2005). Surprisingly, it has been reported that TERT is exported from the nucleus to the cytosol and then gets re-translocated to mitochondria where it protects mitochondrial function and increases resistance against oxidative stress (Ahmed et al., 2008; Haendeler et al., 2009). Similarly, a study has demonstrated that the TERT transferred to mitochondria specifically protects cells from nDNA damage and apoptosis by reducing mtROS levels (Singhapol et al., 2013). Nevertheless, what mechanism is behind the pro-survival function remains elusory and whether the mechanism is linked with telomere homeostasis or a non-telomeric function of TERT is not clear.

### Oxidative stress and accelerated telomere attrition

As is known, the maintenance of telomere homeostasis is a complex mechanism, and telomere shortening, as a biological hallmark of aging, occurs as a natural part of the aging process. However, at present, a robust body of research suggests that telomere length is subject to factors such as oxidative stress, inflammation, behavioral factors, and exposure to carcinogens or radiation (Starkweather et al., 2014; Patel et al., 2017; Barragán et al., 2021). Although plenty of environmental and genic factors are involved in expedited telomere attrition, oxidative stress is presumed to be the most important underlying mechanism (Figure 4B) (Barnes et al., 2019).

In recent years, increasing studies have reportedly shown that oxidative lesion and chronic inflammation either directly or indirectly promote telomere shortening in analyses of human different tissues, mice models, and cell cultures, particularly of vitro cultured cells (Reichert and Stier, 2017; Ahmed and Lingner, 2018; Barnes et al., 2019). Human correlative studies in a review summarized that six of eight population studies reported various oxidative stress markers remarkably related to shorter average telomere length in white blood cells (Reichert and Stier, 2017). Moreover, the majority of degenerative and inflammatory diseases with telomere attrition as mentioned above were parallels with elevated ROS (Zhang J. et al., 2016). Evidence from deficient nfkb1 subunit of the inflammatory gene nuclear factor κB (NF-κB) in mice models demonstrated that chronic inflammation-induced ROS accelerated aging through telomere dysfunction and cell senescence (Jurk et al., 2014). Finally, the mechanism of telomere attrition has been fully researched *in vitro*, clarifying that oxidative stress is a major factor in accelerating telomere loss in a dose-dependent manner (von Zglinicki, 2002; Richter and von Zglinicki, 2007). The later review indicated that oxidative stress or exposure to ROS-producing agents exasperated telomere loss in cultured normal human cells, whereas free-radical scavengers and antioxidants decelerated it and improved replicative lifespan (von Zglinicki, 2002).

There are several mechanisms to exposit how a high level of ROS accelerates telomere dysfunction, among which telomeric DNA damage is the most pervasive and conspicuous theory (Barnes et al., 2019). The following reasons explain why telomere is specifically susceptible to oxidative stress. First, on the account of high guanine content, telomeric DNA is particularly sensitive to oxidative damage and normally forms 8-oxoG, which participates in the acceleration of telomere shortening (von Zglinicki, 2002; Kawanishi and Oikawa, 2004). Oxidative stress can not only damage double stranded DNA but also lead to single-strand DNA breaks which result in incomplete replication of telomere in the next cellular division, consequently, causing more telomere loss (von Zglinicki et al., 2000). Second, due to the protective role of shelterin in telomeres, impaired telomeric DNA by ROS may not effectively activate DDR and make DNA repair proteins into telomeres. Thus, ROS-mediated telomeric regions may have low repair efficiency. Finally, the 3′ overhang of telomere and d-loop are single-stranded whose bases are not merely unprotected by hydrogen bonds unique to double-stranded, but cannot be repaired by base excision repair in the absence of the other complementary chain that offers a template for the impaired DNA re-synthesis (Ahmed and Lingner, 2018). Similarly, the G-quadruplex region, folded from telomeric G-tail, is a highly stable four-stranded helical structure and cannot be repaired *via* base excision repair as the 8-oxoG cannot be excised in quadruplex DNA (Zhou et al., 2013). While Neil3 and NEIL1 DNA glycosylases can remove 8-oxoG from quadruplex DNA, how to repair emerging abasic sites without the complementary stranded part is unclear at present (Zhou et al., 2013, 2015). When the ROS-induced 8-oxoG acts as a substrate for telomere extension, incorporated 8-oxoG influences telomerase activity and terminates 5'-TTAGGG-3' repeat sequences addition, other than perturbing the replication of telomeric DNA (Fouquerel et al., 2016). Strikingly, oxidative damage to telomeric DNA may likewise elongate telomere length by telomerase in certain conditions, nevertheless, present data from cell and animal models accommodate apparent evidence for the predominance of negative effects of ROS on telomere length *in vivo* (Ahmed and Lingner, 2018).

However, given that most of the evidence for the effects of oxidative damage on telomere function comes from cultured cells, oxidative damage has been questioned as a key factor in telomere shortening *in vivo*. A review summarized the limited relative experimental research in this regard and then inferred a conclusion that most experimental results partially or completely supported this view (Reichert and Stier, 2017). On top of that, due to the complexity of ROS that it can destroy multiple cellular components and change cell signaling and gene expression, whether the variations of telomeric length and completeness in oxidative lesion should be attributed to indirect factors or direct damage to the telomeres remains elusive (Barnes et al., 2019; Fouquerel et al., 2019). In the future, more experimental studies on different species are needed to find out the specific mechanism of the oxidative lesion in interfering with telomere length.

### Ang II accelerates senescence *via* oxidative telomere attrition

A previous study(Herbert et al., 2008) *in vitro* showed that the generation of ROS caused by Ang II binding to AT_1_R gave rise to DNA damage, and accordingly induced aging of human vascular smooth muscle cells. Ang II-induced ROS production contributes to cell senescence through two different pathways of DNA damage: stress-induced premature senescence independent of the telomere and accelerated replicative senescence dependent on the telomere. Interestingly, the type of vascular cell senescence appears to depend on the duration of Ang II stimulation (Min et al., 2009). Specifically, cells treated with Ang II for 24 h led to acute stress-induced premature senescence, while cells continually exposed to Ang II over a period of 30 days would accelerate telomere loss and further promote premature replicative senescence. Both senescence pathways were in a dose-dependent manner and ameliorated by catalase or losartan. Also, they shared mutual signaling pathways, i.e., increased p53-p21 axis expression and succeeding hypo-phosphorylation of Rb protein which regulates cell cycle arrest (Toussaint et al., 2000; Herbert et al., 2008). Feng et al. (2011) have demonstrated that Ang II-mediated oxidative stress accelerates senescence by telomere attrition. In this study, treatment of human glomerular mesangial cells with Ang II for 72 h exhibited marked telomere shortening, leading to cell growth arrest, probably *via* P53 and P21 overactivation. Equally, losartan prominently attenuated telomere attrition and cell senescence.

Similarly, two studies (Wilson et al., 2008; Atturu et al., 2010) have found reduced leukocyte telomere length in patients with abdominal aortic aneurysm, which is an age-related vascular disease and can be induced by Ang II *via* medial accretion of macrophages in aortic elastin degradation (Saraff et al., 2003). This case-control study (Atturu et al., 2010) collected data from 190 patients with abdominal aortic aneurysm and 183 controls and found that the average white cell telomere length in abdominal aortic aneurysm patients was significantly shorter compared to controls. Furthermore, in the murine model, TERT deficiency in bone marrow-derived macrophages alleviated Ang II-induced abdominal aortic aneurysm formation (Findeisen et al., 2011).

**Figure 5:**
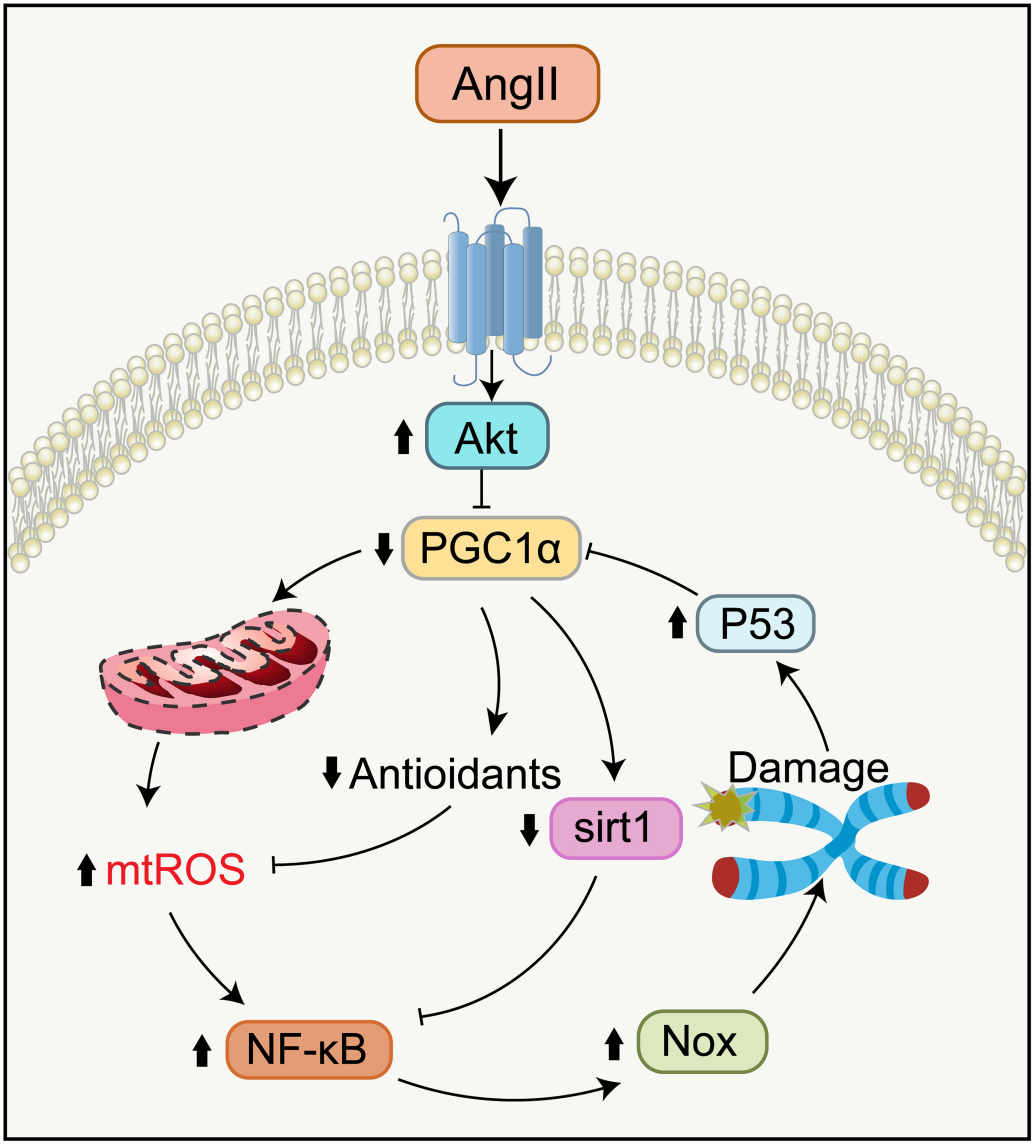
Signaling pathways in telomere attrition and mitochondrial dysfunction during aging. Ang II binding to AT_1_R reduces the function of PGC-1α via activating Akt, causing the compromises of Sirt1 activity and oxidative defense. Telomere attrition mediates mitochondrial impairment by the p53-dependent downregulation of PGC-1α. Impaired mitochondria generate more mtROS which leads to further telomere attrition and replicative senescence by NF-κB-dependent upregulation of Nox. NF-κB, nuclear factor κB.

From molecular signaling pathways, Ang II-induced mitochondrial impairment and telomere attrition are closely related and interact with each other (Figure 5) (Sahin and Depinho, 2010; Chakravarti et al., 2021). Specifically, telomere attrition activates p53, inhibiting PGC1α/β expression, which triggers mitochondrial impairment and diminishes the expression of genes controlling oxidative defense. This signaling pathway of telomere/p53/PGC1α/β/mitochondria leads to escalated levels of mtROS generation and further aggravates guanosine base damage at telomeres. Besides, this increased mtROS activates the NF-κB, raising the expression of Nox, which lowers telomerase activity and expression and drives telomere shortening as well as replicative senescence (Sahin et al., 2011; Salazar et al., 2017; Salazar, 2018).

Overall, Ang II-induced telomere attrition plays an important role in the aging of human cells. Despite many unanswered questions, these findings propose novel mechanisms to directly implicate Ang II in cell senescence and age-associated disorders.

## Conclusions and perspectives

The renin–angiotensin system is involved in a variety of mechanisms regarding tissue and cell senescence and lifespan. Ang II is a critical factor in such senescence, augmenting oxidative damage and inflammation through the AT_1_R. Accumulating evidence indicates that blocking the RAS or applying antioxidants can efficiently restrain organ dysfunction and aging caused by cellular senescence; accordingly, RAS inhibitors and antioxidants hold promise as an anti-aging drug. As detailed in this review, we describe the specific generation and constitution of Ang II-mediated ROS and their biological activities, followed by characterizing the theory of free radicals in aging that posits the gradual accretion of damage inflicted by ROS as an essential driver for aging. Some mechanisms have also been presented to interpret how raised ROS damage mitochondria, change telomeric homeostasis, and therefore lead to aging.

Indeed, these hallmarks during aging are intimately interconnected and develop a new molecular circuit of aging containing the interaction between telomere, p53, and mitochondria (Sahin and Depinho, 2010; Sahin and DePinho, 2012). Research work involving telomerase-deficient mice has uncovered that telomere dysfunction induces functional deterioration of mitochondria. Mitochondrial dysfunction is mediated by repressing PGC-1α/β expression through telomere attrition-induced activation of p53. Remarkably, damaged mitochondria can produce much more ROS and thereby forge a vicious cycle where telomeres may suffer further insult from oxidative stress. Furthermore, TERT can shuttle from the nucleus to mitochondria to protect the nucleus from oxidative damage-inflicted DNA damage and this protective activity is mediated by low levels of ROS. Importantly, mitochondrial dysfunction and fragmentation caused by Ang II *via* increasing PGC-1α acetylation also induce telomere attrition. Mechanistically, dysfunctional mitochondria generate mtROS, activating NF-κB dependent upregulation of Nox that reduces the activity of telomerase and causes telomere attrition and replicative senescence. Nevertheless, understanding their precise causal network is a huge and exciting challenge in future work as the hallmarks co-occur and interconnect with each other during aging.

## Author contributions

ZC and WY designed and outlined the structure and contents of the review. WY and PT created the figures. All authors participated in the writing, revision of the manuscript, and approved the final manuscript.

## Funding

This work was supported by the Construction Project of Capacity Improvement Plan for Chongqing Municipal Health Commission Affiliated Unit (2019NLTS001)-ZS03174, Operating Grant to Chongqing Key Laboratory of Neurodegenerative Diseases (1000013), and Plan for High-level Talent Introduction (2000055).

## Conflict of interest

The authors declare that the research was conducted in the absence of any commercial or financial relationships that could be construed as a potential conflict of interest.

## Publisher's note

All claims expressed in this article are solely those of the authors and do not necessarily represent those of their affiliated organizations, or those of the publisher, the editors and the reviewers. Any product that may be evaluated in this article, or claim that may be made by its manufacturer, is not guaranteed or endorsed by the publisher.
